# Molecular Characterization of the *Alfalfa mosaic virus* Infecting *Solanum melongena* in Egypt and the Control of Its Deleterious Effects with Melatonin and Salicylic Acid

**DOI:** 10.3390/plants10030459

**Published:** 2021-02-28

**Authors:** Ahmed R. Sofy, Mahmoud R. Sofy, Ahmed A. Hmed, Rehab A. Dawoud, Ehab E. Refaey, Heba I. Mohamed, Noha K. El-Dougdoug

**Affiliations:** 1 Botany and Microbiology Department, Faculty of Science, Al-Azhar University, Cairo 11884, Egypt; ahmed_hmed@azhar.edu.eg (A.A.H.); ehabrefaey@azhar.edu.eg (E.E.R.); 2 Virus and Phytoplasma Research Department, Plant Pathology Research Institute, Agricultural Research Center (ARC), Giza 12619, Egypt; rehab_dawood2011@yahoo.com; 3 Department of Biology, Faculty of Science, Jazan University, P.O. Box 114, Jazan 45142, Saudi Arabia; 4 Department of Biological and Geological Sciences, Faculty of Education, Ain Shams University, Cairo 11566, Egypt; hebaebrahem@edu.asu.edu.eg; 5 Botany and Microbiology Department, Faculty of Science, Benha University, Benha 13518, Egypt; nohaeldougdoug@gmail.com

**Keywords:** eggplant, AMV, immunity boosting, RT-PCR, sequencing, antioxidant enzymes, gene expression, oxidative damage

## Abstract

During the spring of 2019, distinct virus-like symptoms were observed in the Kafr El-Sheikh Governorate in Egypt in naturally infected eggplants. Leaves of affected plants showed interveinal leaf chlorosis, net yellow, chlorotic sectors, mottling, blisters, vein enation, necrotic intervention, and narrowing symptoms. The *Alfalfa mosaic virus* (AMV) was suspected of to be involved in this disease. Forty plant samples from symptomatic eggplants and 10 leaf samples with no symptoms were collected. The samples were tested by double antibody sandwich ELISA (DAS-ELISA) using AMV-IgG. Six of the 40 symptomatic leaf samples tested positive for AMV, while, DAS-ELISA found no AMV in the 10 leaf samples without symptoms. The AMV Egyptian isolate (AMV-Eggplant-EG) was biologically isolated from the six positive samples tested by DAS-ELISA and from the similar local lesions induced on *Chenopodium amaranticolor* and then re-inoculated in healthy *Solanum melongena* as a source of AMV-Eggplant-EG and confirmed by DAS-ELISA. Reverse transcription polymerase chain reaction (RT-PCR) assay with a pair of primers specific for coat protein (CP) encoding RNA 3 of AMV yielded an amplicon of 666 bp from infected plants of *Solanum melongena* with AMV-Eggplant-EG. The amplified PCR product was cloned and sequenced. Analysis of the AMV-Eggplant-EG sequence revealed 666 nucleotides (nt) of the complete CP gene (translating 221 amino acid (aa) residues). Analysis of phylogeny for nt and deduced aa sequences of the CP gene using the maximum parsimony method clustered AMV-Eggplant-EG in the lineage of Egyptian isolates (shark-EG, mans-EG, CP2-EG, and FRE-EG) with a high bootstrap value of 88% and 92%, respectively. In addition to molecular studies, melatonin (MTL) and salicylic acid (SA) (100 μM) were used to increase the resistance of eggplant to AMV- infection. Foliar spray with MLT and SA caused a significant increase in the morphological criteria (shoot, root length, number of leaves, leaf area, and leaf biomass), chlorophyll and carotenoid content, antioxidant enzymes, and gene expression of some enzymes compared to the infected plants. On the other hand, treatment with MLT and SA reduced the oxidative damage caused by AMV through the reduction of hydrogen peroxide, superoxide anions, hydroxyl radicals, and malondialdehyde. In conclusion, MLT and SA are eco-friendly compounds and can be used as antiviral compounds.

## 1. Introduction

Plant viral diseases cause severe economic losses due to agricultural production and have hindered sustainable agricultural development globally for a long time [1]. Unlike diseases induced by fungi and bacteria, viral infections are difficult to control once the plants are infected [1,2]. Over recent decades, various strategies have been developed to control viral diseases, including breeding virus-resistant/tolerant cultivars through conventional breeding techniques [3] and using eco-friendly chemical compounds that induce systemic resistance [4,5,6,7].

The eggplant (*Solanum melongena*) is one of the most important vegetable crops in cultivation in Egypt, following potatoes and tomatoes [8]. During their biological cycle, eggplants are very sensitive to many abiotic and biotic stresses [9,10], which can cause substantial crop damage. Therefore, enhancing tolerance to biotic and abiotic stresses is one of the critical goals of eggplant breeding programs. Several viruses were identified as affecting eggplants such as the *Tobacco mosaic virus*, *Tomato mosaic virus*, *Potato virus Y*, *Potato virus X*, *Cucumber mosaic virus*, *Eggplant mottled dwarf nucleorhabdovirus*, and *Eggplant mosaic virus* [11,12]. *Alfalfa mosaic virus* can naturally infect eggplants and cause a great deal of damage to the leaves [13,14]. *Alfalfa mosaic virus* (AMV, genus: *Alfamovirus*, family: *Bromoviridae*) can infect many herbaceous hosts, and some woody plants (150 species in 22 families); it has spread to over 430 species in 51 families of dicotyledonous plants [15]. It is mainly transmitted through seeds, and aphids, specifically *Myzus persicae*, and it can also be spread through the direct transfer of sap from infected hosts to healthy plants [16,17]. The AMV genome is a tripartite single-stranded positive-sense RNA and consists of RNAs 1, 2, and 3, encapsidated into B, M, and T components, respectively [18]. RNAs 1 and 2 encode the replicase subunits 1a, and 2a, respectively [19]. RNA 3 encodes the movement protein [20] and viral coat protein, expressed from a subgenomic RNA 4 [19].

Chemical priming may be considered a timely and successful management technique to induce plants’ resistance/tolerance to viruses. Several eco-friendly compounds that are considered non-toxic, biodegradable, and biocompatible oligo chitosans, such as chitin and chitosan, can be used to induce the resistance of plants to viruses [21,22,23]. Melatonin (MLT, N-acetyl-5 methoxytryptamine), an indoleamine molecule, is regarded as a useful alternative tool to enhance biotic and abiotic stress defense for plants and has been considered a good antioxidant for reactive oxygen and nitrogen species (ROS/RNS) over the last two decades [24]. The major mechanism that facilitates melatonin-induced biotic stress in plants is the up-regulation of defense genes, thickening of the cell wall, ROS scavenging, and NO production [25,26]. These regulatory improvements help cope with unfavorable conditions, assuming reinforcement against plant stress. Moreover, MLT enhances physiological processes such as germination, photosynthesis, primary and secondary metabolism, and plant hormones regulation [27,28]. Furthermore, the disease severity and concentration of *Tobacco mosaic virus* (TMV) in infected *Nicotiana glutinosa* and *Solanum lycopersicum* seedlings decreased after foliar spray with 100 µM melatonin twice [29]. Additionally, MLT has been considered as a therapeutic indole for combating viral diseases, such as SARS (severe acute respiratory syndrome) and WNV (*West Nile virus*) [30].

Salicylic acid (SA) is considered a plant hormone and phenolic compound and acts as a central signaling network regulator in environmental and pathogen infection stress conditions in plants [2,31,32]. SA participates directly in plant growth, induction of flowers, ion uptake, yield, and the improvement of chlorophyll pigment content, photosynthetic rate, and the activity of certain essential enzymes [33]. The initial report on salicylate’s role as a disease-inducing agent was discovered against TMV on tobacco in 1979 [34]. Furthermore, SA can increase the resistance against *Tomato yellow leaf curl virus* (TYLCV) by modulating the expression of genes that encode ROS scavenging, alter the function of resistance enzymes, and produce systemically acquired resistance to pathogenesis-related genes [35].

Plants possess a range of active defense apparatuses that can be actively expressed in response to biotic stresses (pathogens). If stimulus triggers defense mechanisms before infection by a plant pathogen, the disease can be reduced [2]. Induced resistance is a state of enhanced defensive capacity developed by a plant when appropriately stimulated. Systemic acquired resistance (SAR) and induced systemic resistance (ISR) are two forms of induced resistance wherein plant defenses are preconditioned by prior infection or treatment that results in resistance against subsequent challenge by a pathogen. Plant pathogens or chemical compounds cause systemic acquired resistance (SAR), and their regulation is based on salicylic acid (SA). A number of pathogenesis-related proteins (PRs) play important roles as anti-pathogenic agents such as peroxidase and chitinase [3]. Induced systemic resistance (ISR) is induced by plant microbes that promote growth, and its regulation is based on jasmonic acid and ethylene [2].

The aims of the study were to document the association of AMV with eggplant disease syndrome, to study the genetic variability of Egyptian AMV isolate recovered from eggplants, to assess the full CP homology between this isolate and other AMV isolates reported in Egypt and elsewhere in the GenBank database and to induce the resistance of eggplants to virus infection through the use of eco-friendly compounds like MLT and SA.

## 2. Results

### 2.1. Symptomatology, Examination and Biological Purification of AMV-Eggplant Egyptian Isolate

**Figure 1:**
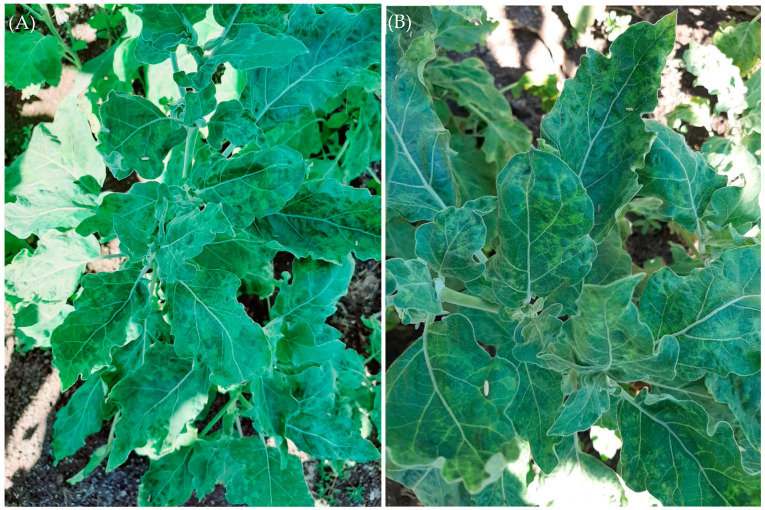
Eggplant (*Solanum melongena*) leaves showing different virus-like symptoms such as interveinal leaf chlorosis, net yellow, chlorotic sectors, mottling, blisters, vein enation, necrotic intervention, and narrowing symptoms. Photo (**B**) is zoomed from photo (**A**).

During the spring of 2019, distinct virus-like symptoms were observed in the Kafr El-Sheikh Governorate in Egypt in naturally infected eggplant. Leaves of the affected plants showed interveinal leaf chlorosis, net yellow, chlorotic sectors, mottling, blisters, vein enation, necrotic intervention, and narrowing symptoms (Figure 1). *Alfalfa mosaic virus* (AMV) was suspected to be been involved in the disease. Forty plant samples were collected from symptomatic eggplants as well as, 10 leaf samples from plants with no symptoms growing nearby. DAS-ELISA was used to test the samples for the presence of AMV. Six of the 40 symptomatic leaf samples were positive for AMV, while, DAS-ELISA found no evidence of AMV in the samples from the 10 leaf samples without symptoms.

**Figure 2:**
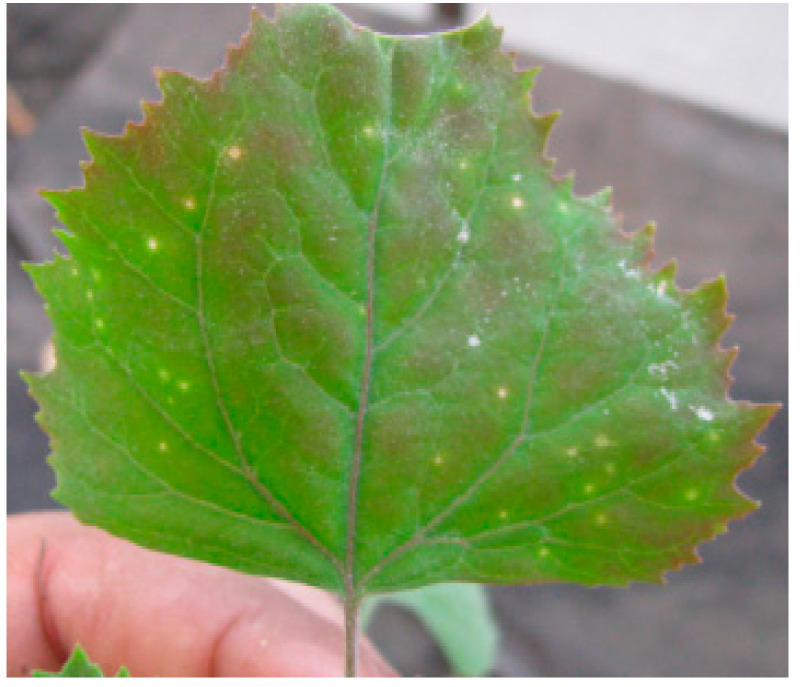
*Chenopodium amaranticolor* mechanically inoculated with AMV-Eggplant-EG.

*Alfalfa mosaic virus* Egyptian isolate (AMV-Eggplant-EG) was isolated from six positive samples tested by DAS-ELISA and from the similar local lesions induced on *Chenopodium amaranticolor* (Figure 2) and then re-inoculated in healthy *Solanum melongena* as a source of AMV-Eggplant Egyptian isolate and confirmed by DAS-ELISA.

### 2.2. Molecular Characterization of AMV-Eggplant Egyptian Isolate

#### 2.2.1. AMV-Eggplant-EG Coat Protein Gene Amplification and Sequencing

RT-PCR amplification of the coat protein (CP) gene of the AMV-Eggplant Egyptian isolate was performed on the total RNA isolated from infected *Solanum melongena*. Using the AMV-F2/AMV-R2 primer set, it was designed to amplify the entire AMV/CP gene, generating an amplicon of 666 bp. The specific primers have not amplified viral cDNA from healthy plants (control). The amplified PCR product was cloned and sequenced where the complete nucleotide sequence of the amplified PCR fragment of the AMV-Eggplant-EG/CP gene was determined. The relationship with the CP gene sequences of different AMV strains available at GenBank was determined through a multiple sequence alignment and phylogenetic analysis. The sequenced region contained a single open reading frame, consisting of 666 nucleotides (nt)potentially coding for 221 amino acids (aa).

#### 2.2.2. Viroinformatics Analysis of AMV-Eggplant-EG/CP Gene

**Figure 3:**
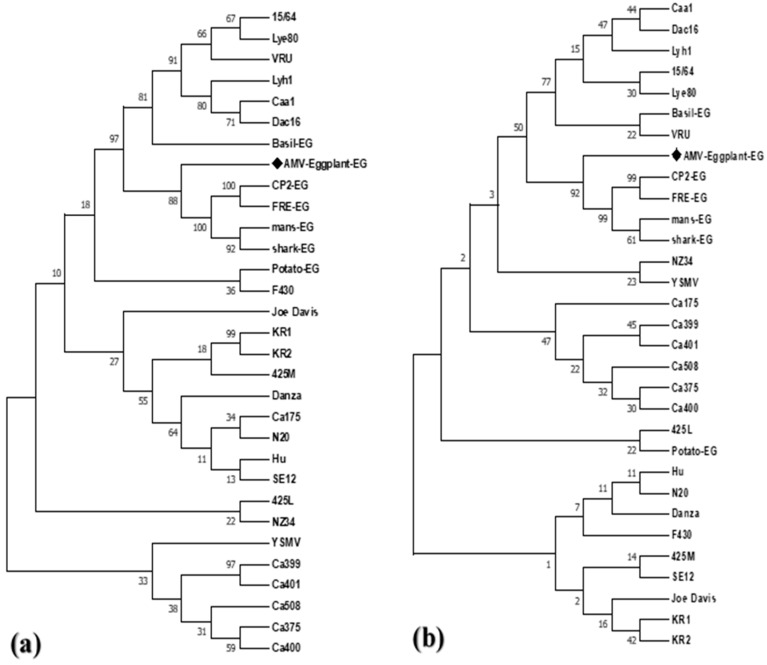
Phylogenetic tree AMV-Eggplant-EG isolates and 30 AMV isolates available in GenBank produced using the Maximum Parsimony method (numbers representing bootstrap percentage values based on 1000 replicates are shown next to the relevant branches), based on the complete nucleotide sequences (**a**) and complete amino acids (**b**) of the CP gene.

**Figure 4:**
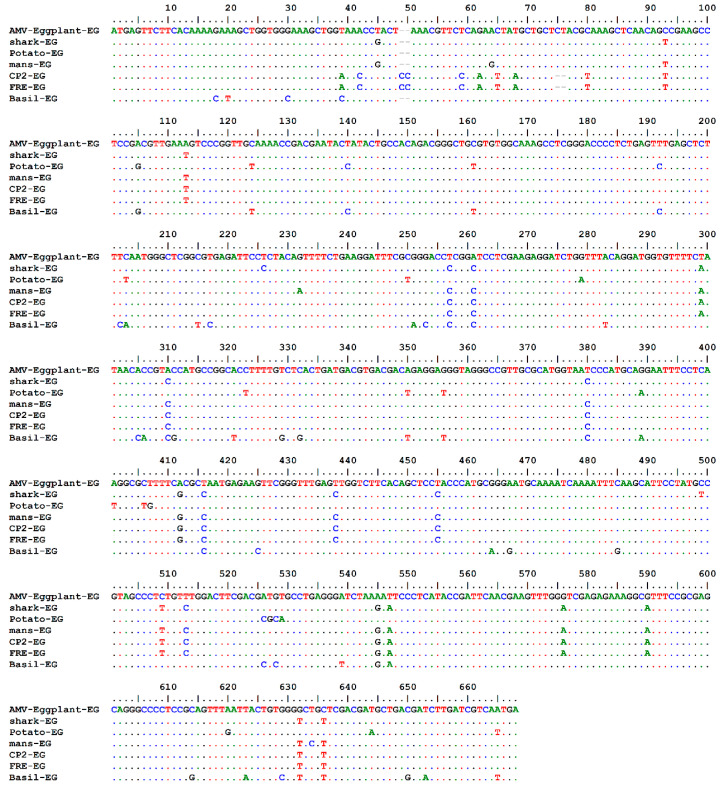
Nucleotide sequence alignment of the AMV-Eggplant-EG isolate coat protein with Egyptian AMV isolates.

**Figure 5:**
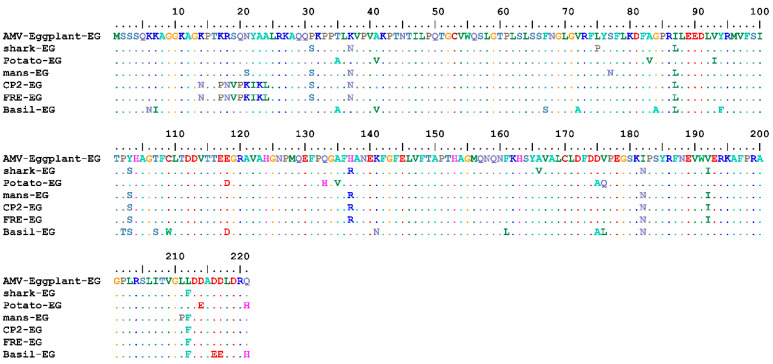
Amino acid sequence alignment of the AMV-Eggplant-EG isolate coat protein with Egyptian AMV isolates.

Complete AMV-Eggplant-EG/CP gene nt and deduced aa sequences were multiply aligned using the ClustalW program with minor manual adjustments to compare with the corresponding sequences of the full CP gene of the various AMV isolates reported in GenBank, determining nt identities from 92.94% to 96.70% and aa identities from 89.59% to 95.48%. Pairwise similarity has shown that the AMV-Eggplant-EG/CP was closely related to AMV-Shark-EG/CP (accession no. LN846978) in nt 96.70% and aa with 95.48% (Table 1). The highest nt identity of 96.70% was found with the Egyptian isolates shark-EG and Potato-EG (LN846978 and HQ288892, respectively). The lowest nt identity of 92.94% was identified in the Egyptian isolate Basil-EG (MH625710). On the other hand, the highest aa identity of 95.48% was found in the Egyptian isolate shark-EG (LN846978) and Korean isolate KR2 (AF294433). The lowest aa identity of 89.59% was determined in the Egyptian isolate Basil-EG (MH625710). The nt and aa sequences of the CP gene of the AMV-Eggplant-EG, along with these retrieved from GenBank (30 isolates), were used to construct a maximum parsimony phylogenetic tree (Figure 3). AMV-Eggplant-EG was clustered in the lineage of Egyptian isolates (shark-EG, mans-EG, CP2-EG, and FRE-EG) with high bootstrap value of 88% and 92%, respectively (Figure 3). AMV-Eggplant-EG isolate coat protein alignment of the nt and aa sequences with Egyptian AMV isolates was demonstrated in (Figure 4 and Figure 5). There was a total of 668 and 221 positions in the final dataset for nucleotide and amino acid sequences, respectively. A total of 79 and 45 variable sites were found in Egyptian AMV isolates nucleotide and amino acids, respectively, including the gaps where 38 and 22 were parsimoniously informative nucleotide and amino acids, respectively. In addition, 41 and 23 were single sites (Figure 4 and Figure 5).

### 2.3. Systemic Protection against Alfalfa mosaic virus (AMV) in Eggplant

**Figure 6:**
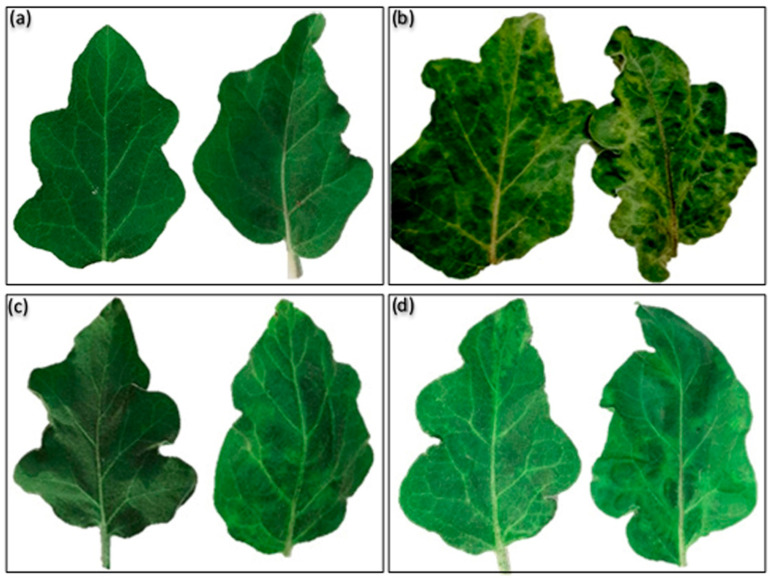
Mock inoculated (**a**) and *Alfalfa mosaic virus* symptoms in challenge control (**b**) and eggplant foliar sprayed with MLT (**c**) and SA (**d**) at 21 days after inoculation.

AMV symptoms, including mottling, net yellow, blisters, leaf narrow, leaf distortion, vein enation, and necrotic intervention disease severity of 90%, were observed for the infected eggplants (Figure 6b and Table 2) compared to mock inoculated eggplants (Figure 6a). The protective antiviral activity of MLT and SA against AMV was evaluated. The results show that foliar spraying of MLT and SA substantially reduced the virus concentration, the percentage of infection with the virus, and the disease severity (DS), compared to challenge control (ChC) (Figure 6b–d and Table 2).

### 2.4. Physiological and Biochemical Studies

#### 2.4.1. Changes in Plant Growth

**Figure 7:**
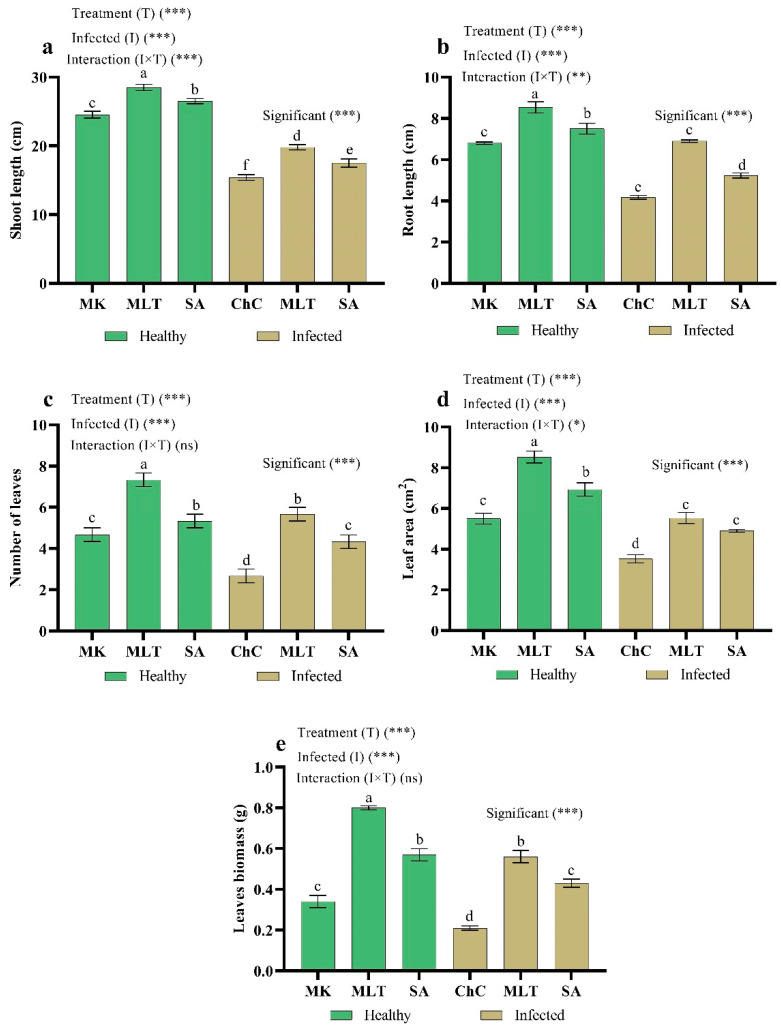
Effect of foliar spraying of MLT and SA on plant growth. Shoot length (**a**), root length (**b**), number of leaves (**c**), leaf area (**d**), leaf biomass (**e**) of eggplant under mock inoculation (MK), and AMV infection (challenge control, ChC). According to Fisher’s test, the different letters (a–f) are significantly different between treatments at the 0.05 level. Vertical bars represent the means of 10 independent determinations ± standard error (SE). *, **, ***, and ns (non-significant) indicate significant and highly significant differences according to two-way analysis of variance (ANOVA) with a Fisher’s post hoc test.

The AMV infection caused a drastic decrease in shoot length (37.2%), root length (28.7%), number of leaves (42.8%), leaf area (35.8%), and leaf biomass (38.2%) on eggplants as compared with mock inoculated plants (Figure 7). In addition, eggplants challenged with AMV and treated with MLT, and SA recorded substantial increases in all morphological parameters compared to the challenge control plants. MLT and SA resulted in the highest values in all the morphological parameters in healthy and infected plants. In addition, the highest increases in shoot length (28.4%), root length (65.5%), number of leaves (112.4%), leaf area (56.7%), and leaf biomass (166.7%) were recorded in plants treated by MLT as compared to the challenge control plants (Figure 7).

#### 2.4.2. Changes in Photosynthetic Pigments

**Figure 8:**
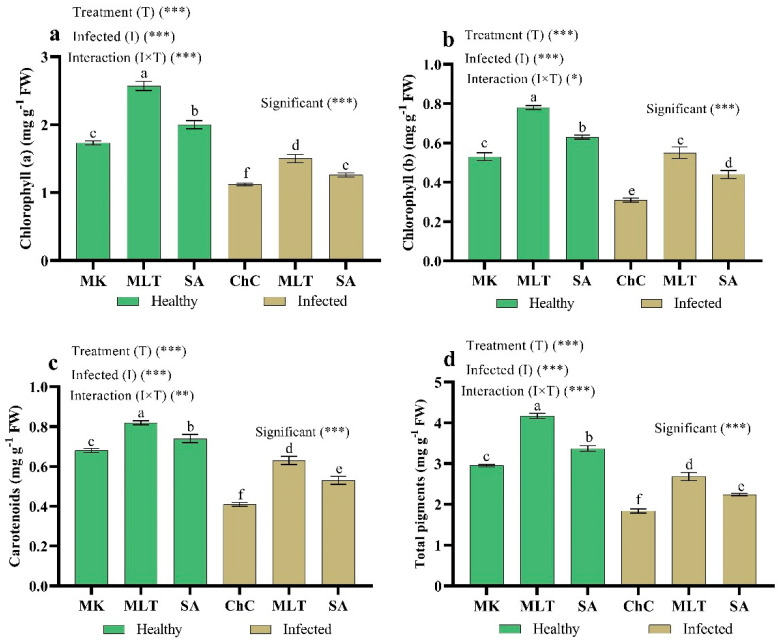
Effect of foliar spraying of MLT and SA on chlorophyll a (**a**), chlorophyll b (**b**), carotenoids (**c**), and total pigments (**d**) content in leaves of eggplant under mock inoculation (MK) and AMV infection (challenge control, ChC). According to Fisher’s test, the different letters (a–f) are significantly different between treatments at the 0.05 level. Vertical bars represent the means of three independent determinations ± standard error (SE). *, ** and *** indicate significant, highly significant difference according to two-way ANOVA with a Fisher’s post hoc test.

The data in Figure 8 show that infected eggplants with AMV caused a significant decrease in chlorophyll a (35.3%), chlorophyll b (41.5%), carotenoid (39.7%), and total chlorophyll content (37.6%) relative to mock inoculated plants. In addition, MLT and SA not only counteracted the drastic influence of AMV on chlorophyll concentration but induced a considerable stimulating impact of chlorophyll assimilation compared to the challenge control plants. The most effective treatment for enhancing chlorophyll content was MLT, which increased the content of Chl a by 33.9%, Chl b by 77.4%, carotenoid by 53.7%, and total chlorophyll by 45.7% in the leaves of eggplants over the challenge control plants (Figure 8).

#### 2.4.3. Changes in ROS Damage

**Figure 9:**
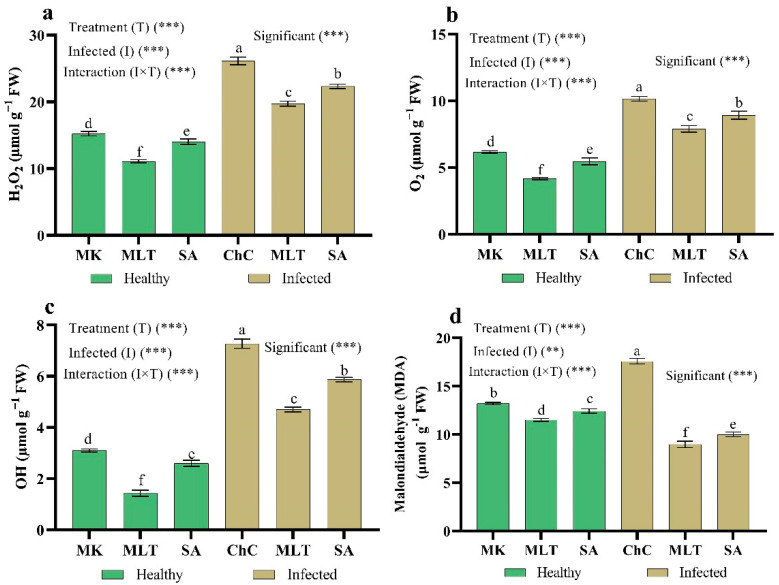
Effect of foliar spraying of MLT and SA on ROS content (H_2_O_2_ (**a**), O_2_ (**b**), OH (**c**), and malondialdehyde (MDA) (**d**)) content in leaves of eggplants under the mock inoculation (MK) and AMV infection (challenge control, ChC). According to Fisher’s test, the different letters (a–f) are significantly different between treatments at the 0.05 level. Vertical bars represent the means of three independent determinations ± standard error (SE). ** and *** indicate highly significant difference according to two-way ANOVA with a Fisher’s post hoc test.

One of the harmful effects of viral infection is oxidative damage to cell membranes. To examine the role of MLT and SA in oxidative damage, we next measured the reactive oxygen species production and malonaldehyde (MDA) content in eggplant leaves infected with AMV. H_2_O_2_, O_2_^−^ OH, and MDA contents increased by 71.6%, 64.8%, 134.5%, and 33.1%, respectively, in challenge control plants compared to the mock inoculated plants (Figure 9). Meanwhile, treatment with MLT and SA significantly decreased the H_2_O_2_ content (24.5% and 14.5%), O_2_^−^ (22.3% and 12.2%), OH (35.6% and 19.3%), and MDA content (49.0% and 43.1%) as compared to the challenge control plants, respectively (Figure 9).

#### 2.4.4. Changes in Secondary Metabolites and Salicylic Acid Content

**Figure 10:**
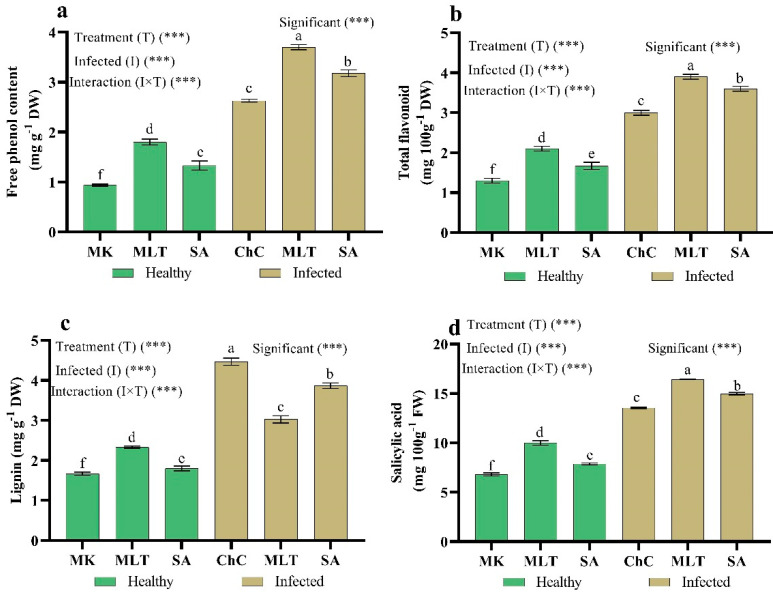
Effect of foliar spraying of MLT and SA on phenol content (**a**), flavonoids content (**b**), lignin content (**c**), and salicylic acid content (**d**)) content in leaves of eggplant under mock inoculation (MK) and AMV infection (challenge control, ChC). According to Fisher’s test, the different letters (a–f) are significantly between treatments at the 0.05 level. Vertical bars represent the means of three independent determinations ± standard error (SE). *** indicates highly significant difference according to two-way ANOVA with a Fisher’s post hoc test.

To clarify MLT and SA’s role in eggplants infected with AMV, we assessed the secondary metabolites, lignin, and endogenous SA production. The results revealed that total phenol, flavonoids, lignin, and endogenous SA content rose considerably by about 180%, 130.8%, 167.7%, and 25% respectively, in eggplants inoculated with AMV compared to non-inoculated plants (Figure 10). Moreover, the foliar spraying of challenge control eggplants with MLT and SA led to a higher accumulation of total phenol, flavonoids, lignin, and endogenous SA content compared to challenge control eggplants (Figure 10).

#### 2.4.5. Changes in Antioxidant Enzymes

**Figure 11:**
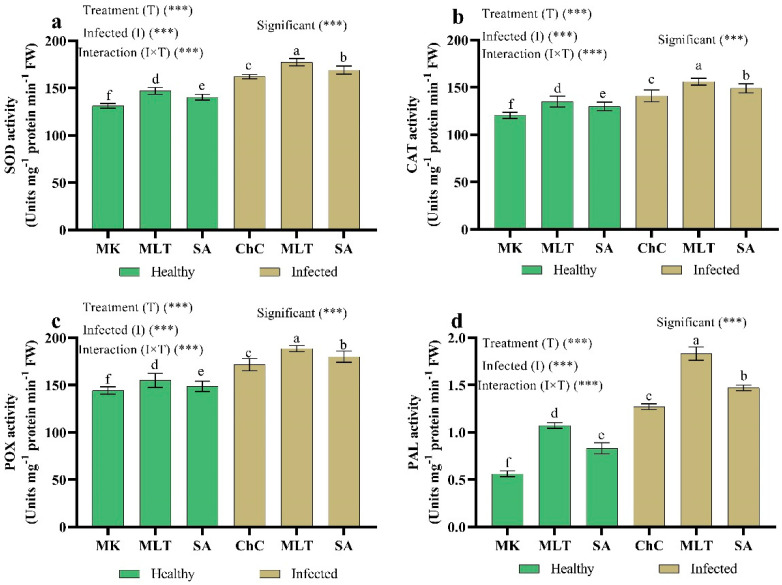
Effect of foliar spray by MLT and SA on enzymatic antioxidants content in leaves of eggplant under mock inoculation (MK) and AMV infection (challenge control, ChC). SOD (**a**), CAT (**b**), POX (**c**), and PAL (**d**). According to Fisher’s test, the different letters (a–f) are significantly different between treatments at the 0.05 level. Vertical bars represent the means of three independent determinations ± standard error (SE). *** indicates a highly significant difference according to two-way ANOVA with a Fisher’s post hoc test.

Eggplant infection with AMV was more effective in terms of increasing the activity of all antioxidant enzymes studied, including superoxide dismutase (SOD), catalase (CAT), peroxidase (POX), and phenylalanine ammonia-lyase (PAL), when compared to the mock inoculated plants (Figure 11). In addition, treatment with MLT and SA in challenge plants with AMV stimulated the activity of SOD (9.7% and 4.3%), CAT (10.6% and 5.7%), POX (9.9% and 4.3%), and PAL (49.9% and 15.6%) as compared to the challenge control plants, respectively (Figure 11).

#### 2.4.6. Changes in Gene Expression

**Figure 12:**
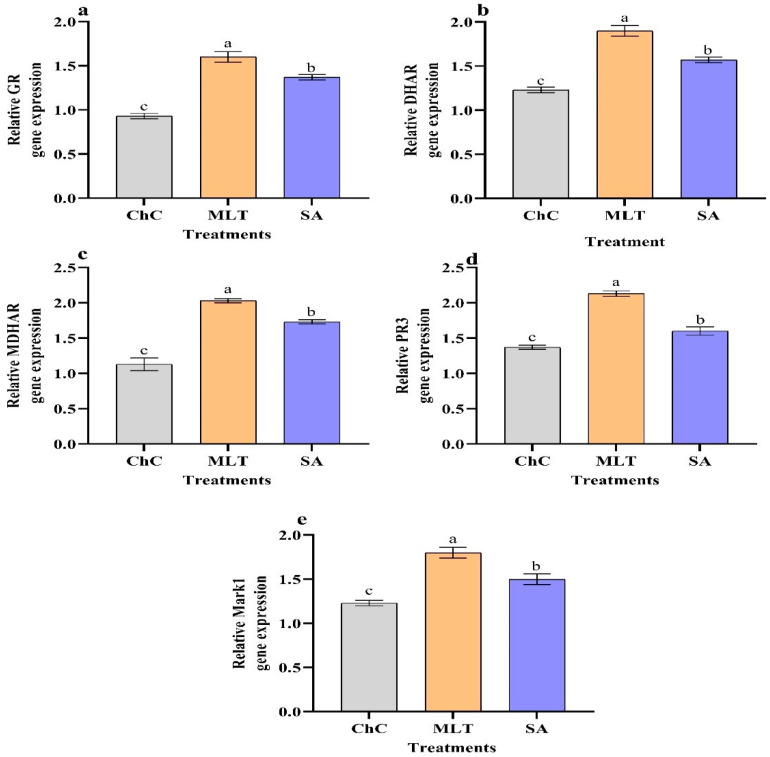
Effect of foliar spraying of MLT and SA on the gene expression of relative Glutathione reductase (GR) (**a**), Dehydroascorbate reductase (DHAR) (**b**), Monodehydroascorbate reductase (MDHAR) (**c**), Chitinase (PR3) (**d**), and mitogen-activated protein kinase (MPK1) (**e**) in leaves of eggplant under AMV infection and challenge control (ChC; untreated CMV-infected plants). According to Fisher’s test, the different letters (a–c) are significantly different between treatments at the 0.05 level. Vertical bars represent the means of three independent determinations ± standard error (SE).

Significant increases in the relative expression levels of GR, DHAR, MDHAR, PR3, and MPK1 were seen in infected eggplants treated with MLT and SA compared to the challenge control plants (Figure 12). Compared to the challenge control, a significant up-regulation of GR, DHAR, MDHAR, PR3, and MPK1 with relative expression levels representing 72%, 54.5%, 79.5%, 55.5%, and 22% increases were detected in plants treated with MLT, respectively (Figure 12).

## 3. Discussion

During the spring of 2019, the eggplants grown in an open field (Kafr El-Sheikh Governorate, Egypt) showed interveinal leaf chlorosis, net yellow, chlorotic sectors, mottling, blisters, vein enation, necrotic intervention, and narrowing symptoms. The presence of *Alfalfa mosaic virus* (AMV) in individual plants was serologically tested by DAS-ELISA. Six of the 40 symptomatic leaf samples were positive for AMV, while, DAS-ELISA found no evidence of AMV in the 10 leaf samples without symptoms. *Alfalfa mosaic virus* Egyptian isolate (AMV-Eggplant-EG) was isolated from the six positive samples and from the similar local lesions induced on *Chenopodium amaranticolor* and then re-inoculated in healthy *Solanum melongena* as a source of AMV-Eggplant Egyptian isolate and confirmed by DAS-ELISA. AMV is considered one of the most economically important viruses, in that it is a widespread, common virus that infects various plants and consists of many strains that differ in symptomatology between different hosts [2,36]. Several reports have described the natural occurrence of AMV in *Solanum melongena* from India, Turkey, and Saudi Arabia [13,14,37]. The size of the RT-PCR product of AMV-Eggplant-EG infected *Solanum melongena* tissue was identical to that of the 666 bp AMV/CP gene using the AMV-F2/AMV-R2 primer set designed to amplify the entire AMV/CP gene [38]. However, the specific primer pair did not amplify viral cDNA from extracts of uninfected *Solanum melongena*.

In order to classify sequences and variability with the 30 worldwide isolates available in GenBank, the complete CP gene sequence (666 bp) of an isolate of AMV-Eggplant-EG RT-PCR amplified fragment was determined. The maximum parsimony phylogenetic tree of the nucleotide and deduced amino acid sequences showed that AMV-Eggplant-EG clustered in the lineage of Egyptian isolates (shark-EG, mans-EG, CP2-EG, and FRE-EG) with a high bootstrap values of 88% and 92%.

Treatment with MLT or SA substantially reduced the virus concentration and the disease severity in the symptomatic plants’ leaves (Table 2). MLT and SA decreased the AMV concentration that was detected by DAS-ELISA. The reduction in virus concentrations may be due to the increase in the POX enzyme, which is known to catalyze the final polymerization step of lignin synthesis and is directly associated with the increased ability of systemically protected tissues to lignify [39] and help in the defense responses against viral infection. Also, during pathogen attack in plants, AS treatment, including H_2_O_2_ accumulation, can act with at least one of two mechanisms against pathogens: H_2_O_2_ can act directly by killing the pathogen and H_2_O_2_ also hinders the penetration of plants by microorganisms [2]. It contributes to cell wall stiffening by facilitating peroxidase reactions catalyzing intra and inter-molecular cross-links between the structural components of cell walls and lignin polymerization [40]. In addition, the production of ROS can reinforce plant cell walls through cross linking reactions of lignin and protein. ROS are toxic agents against either the host plant cells, with the development of a hypersensitive response and systemic acquired resistance (SAR), or against pathogens, killing them or stopping their growth and development. Moreover, ROS are considered secondary messengers in signaling routes leading to the activation of plant defense related genes [4,40]. Also, in this work, an increase in the level of phenolics and flavonoids with either virus infection or MLT and SA treatments was observed. Flavonoids mainly act as antioxidants, to prevent viral binding and penetration into cells and to trigger host cell self-defense mechanisms [41]. This supports a role of SA in the induction of antioxidants and enhanced resistance. Moreover, the accumulation of SA after treatment with SA and MLT can be dramatically induced in plants when challenged by various pathogens, including viruses [29]. An increased level of SA is necessary for plants to acquire resistance to viral infection by interfering in the major stages of the virus cycle: replication, cell-to-cell movement, and long-distance movement [29].

In this respect, Sudhakar, et al. [42] found that SA accumulation after *Cucumber mosaic virus* (CMV) inoculation contributes to boosting the activity of enzymes such as PAL or POX, helping with the plant’s resistance to viral infection. SA allows for strength mechanisms like the development of phytoalexins, proteinase inhibitors, cell wall strengthening, and lignification. Also, the use of SA on tobacco improved resistance to CMV, and the inhibition of virus movements, demonstrated systemic resistance [43]. Moreover, the *Potato virus Y* (PVY) and *Bean yellow mosaic virus* (BYMV) concentration and infection percentages decreased when the plants were sprayed with SA. This reduction may be due to the suppressed virus replication, reduced virus accumulation, and virus entry into the vascular system via treatment [44,45].

Moreover, treatment with MLT helped with resistance to *Apple stem groove virus* (ASGV) due to encouraging the rapid extension of shoot tips to avoid infection with the virus, reducing the virus amount in the shoot tips and inhibiting viral movement in the shoot tips [30]. In the TMV-infected *Solanum lycopersicum* seedlings, the exogenous use of MEL has substantially increased SA levels. Therefore, increased SA levels can be seen as one of the MEL-mediated virus resistance mechanisms [29].

One effect of the application of MLT and SA is an improvement in plant growth under normal conditions (Figure 7), as well as in plants under viral stress, indirectly due to their strong anti-pathogenic activity and explicitly due to (i) the stimulation of biosynthesis of phytohormones, (ii) the stimulation of soil nutrient absorption and solubilization, and (iii) the stimulation of root hardness and growth [4]. Also, MLT and SA are considered multifunctional plant-growth regulators. They help alleviate the oxidative damage from various stresses [46] and increase gene expression involving cell division, cell expansion, photosynthesis, metabolism, and hormonal balance [27,47,48].

In eggplants infected with AMV, the photosynthetic pigment content was considerably reduced compared to mock inoculated plants (Figure 8). Similar data indicate that CMV infection decreased the chlorophyll content in cucumber plants [4,7,49]. The content of chlorophyll in plants infected with the virus may be reduced due to activating certain cell enzymes such as chlorophyllase [50] or the virus’s effect on pigment synthesis, mineral uptake, and transport [51]. Additionally, applied MLT and SA increased the chlorophyll and carotenoid contents in the leaves of infected plants compared to the infected plants without treatments. Under pathogen infection, the defense function of MLT in chlorophyll can be due to its antioxidant ability and the inhibition of up-regulation in certain senescence-associated genes [52]. In particular, MLT can boost the activity and enhance the photosynthetic carbon assimilation of Calvin cycle enzymes [53]. It was found that an increase in the photosynthesis rate of SA-treated plants could be due to metabolic changes at the level of chloroplasts such as photosystem efficiency II, enzyme activity Rubisco, and the carbon-reducing supply of ATP and NADPH [54]. Moreover, SA improves membrane permeability, making it easy to absorb and use mineral nutrients such as Mg and Fe, which are essential in the biosynthesis of chlorophyll and transport of assimilates [55].

Like other biotic stresses, viral inoculation provokes oxidative stress in different ways. Modified stomatal conductance, disrupted photosystem activity and altered enzymatic activities of cytosol, chloroplast, mitochondrial, or other ultrastructural organs are the common reasons for viral-induced oxidative stress [4,5,56,57,58]. In the present study, eggplants inoculated with AMV showed a highly significant increase in the contents of H_2_O_2_, O_2_^−^, OH, and MDA which are indicators of oxidative stress. Our findings were consistent with those of Xi, et al. [59], who found that synergistic CMV and TNV infections led to higher MDA and H_2_O_2_ rates and lower catalase activity than those individually infected. The accumulation of H_2_O_2_ in host cells plays a significant role in enhancing virus resistance in plants [60]. MLT and SA foliar spraying decreased the H_2_O_2_, O_2_^−^, OH, and MDA, compared to AMV stress alone, and in this case, the lowest reduction of these oxidative stress markers has been documented for 100 μM MLT. MLT and SA have been proven to up-regulate the content of different non-enzymatic antioxidants in addition to the activity of enzymes of the antioxidant defense system under stress conditions, which alleviates the oxidative stress induced by other biotic stresses [61].

Phenolic, flavonoid, lignin, and endogenous SA content are dramatically increased in eggplants infected with AMV alone or treated with MLT and SA (Figure 10). High amounts of phenolic compounds can result in the higher rigidity of the host cell walls through lignin and suberin synthesis, which is regarded as a physical obstacle to propagating viruses [42,62]. Furthermore, phenols such as SA have high antifungal activity and help protect host plants against a fungal pathogen’s infestation [63,64,65]. Through secondary metabolic pathways, plants generate a number of metabolites that work mostly in plant defense responses to pathogen infection and environmental stresses [66,67,68,69,70,71]. Previous studies have shown that CMV can boost phenol and flavonoid concentration in leaves [4]. In addition, a proteomic analysis revealed that MLT can also affect the biosynthesis of flavonoids [28].

In addition, the antioxidant enzymes play essential roles in counteracting the effects of AMV infection. This has become evident through the comparatively increased activity of enzymes that scavenge ROS in cucumber plants [72]. The results of the current study (Figure 11) have supported this finding. The antioxidative enzymes assayed in the present work, such as SOD, CAT, POX, and PAL, play a unique role in terms of mitigating the effects of oxidative stress stimulated by AMV infection. More broadly, TMV and *Tomato mosaic virus* (ToMV) inoculated *Nicotiana glutinosa* seedlings demonstrated improved POD and CAT activity compared with uninfected seedlings [73]. Because SOD is an effective O^2–^ scavenger, it is the plant’s first defense against ROS [74,75], demonstrating the SOD’s defensive role in biological systems. Moreover, POX and CAT may be considered radical scavengers and catalyzed H_2_O_2_ producing H_2_O and O_2_ [76,77]. Thus, the rise in POX may be thought to lead to oxidative stress in systemic interactions between the plant and virus. Up-regulated peroxides may also reduce growth and malformation in virus-infected plants by oxidizing indole-3-acetic acid [78]. Higher amounts of ROS-related enzymes have also been observed in bean plants infected with the *White clover mosaic virus* [79]. Our results show that the activity of all enzymes rose under AMV infection and further increased with MLT and SA treatment, especially MLT. SA plays an essential role in ROS scavenging by boosting the activity of SOD, POX, and APX in tomato plants inoculated with TYLCV [35]. Also, SA-induced higher PAL amounts led to an improved build-up of phenolic compounds and/or antimicrobials after infection with *Potato virus X* [31].

The expression of pathogenesis-related genes like GR, DHAR, MDHAR, PR3, and MPK1 was greater in infected eggplants foliar sprayed with MLT and SA (Figure 12). Similar findings are mentioned by Li et al. [35], who observed that SA could up-regulate gene expression and control TYLCV resistance in tomato plants. Also, MLT increased the gene expression of chitinase (PR3) [80], which has a crucial function in that it reduces lesion expansion and inhibits pathogen growth [81,82]. More broadly, the effects of MLT on biotic stress are a combined consequence of increases in endogenous hormone, antioxidant enzymes, and PR3 expression [83].

In addition, mitogen-activated protein kinase (MAPK/MPK) cascades are strongly preserved signaling modules that translate cell surface signals into cellular specific targets that involve plant growth, development, and biotic and abiotic stress responses [84,85]. Also, SA-treated *Solanum melongena* seedlings after being infected with *Verticillium dahliae* caused an increment in MPK gene expression, triggering the prevailing biosynthetic pathway of SA, based on iso-chorismate (ICS). The biosynthesized SA also controls the expression of stress and defense genes such as PR proteins, which results in systemic resistance. *Solanum melongena* resistance to *Verticillium* host pathogens may be supported by protective response gens (β-1,3-glucanase and chitinase) and growth and developmental participants (IAA27, MPK1, and GPX) [86]. In this regard, Pacheco et al. [54] and Hackmann et al. [87] found that the exogenous application of SA has induced the expression of several defensive genes encoding various secondary metabolic enzymes into bioactive compounds such as phenolics under pathogen infection. PR-2 and PR-3 are essential protein groups, acting on their own or in combination against fungal infection and viruses [4,5,31].

## 4. Materials and Methods

### 4.1. Sample Collection and Virus Detection

Naturally infected eggplant (*Solanum melongena*) with virus-like symptoms were collected from Kafr El-Sheikh Governorate, Egypt. The *Alfalfa mosaic virus* (AMV) was suspected of involvement in this disease. There were 40 plant samples from symptomatic eggplants, and 10 leaf samples with no symptoms were also collected. DAS-ELISA was used to analyze the samples as described by Clark and Adams [88] using AMV polyclonal antibody.

### 4.2. Isolation and Propagation of Virus

The inoculum of infectious sap was prepared by grinding the DAS-ELISA positive leaf samples in 0.1 M phosphate buffer, (pH 7.2, 1:2 containing 0.3% β-mercaptoethanol) in a sterilized mortar and pestle. A single local lesion technique was used for the biological isolation of AMV, according to Noordam [89], where infectious sap was inoculated in *Chenopodium amaranticolor* as a local lesion host. Control plants were inoculated with phosphate buffer. The inoculated plants and control were kept under insect-proof greenhouse condition.

### 4.3. Total RNA Extraction and RT-PCR Amplification of AMV/CP Gene and Sequencing

According to the manufacturer’s instructions, total RNAs were isolated from healthy and infected *Solanum melongena* plants with AMV-Eggplant Egyptian isolate (AMV-Eggplant-EG), using a QIAamp RNA isolation kit. The AMV-F2 and AMV-R2 oligonucleotide primers were designed to amplify the AMV/CP gene according to Xu and Nie [38]. RT-PCR was performed according to Xu and Nie [38], where the mixtures were incubated at 94 °C for 2 min, followed by 35 cycles, with final incubation at 72 °C for 7 min, followed by 4 °C. The PCR-product was purified using a QIAquick PCR purification kit. The purified amplicon was then cloned into a pGEM-T Easy Vector in DH5 *Escherichia coli* competent cells according to the manufacturer’s instructions and directly sequenced using the same primer pair using RT-PCR. Data were analyzed using FinchTV^TM^ version 1.4.0 software of sequencing analysis. The CP gene nucleotide sequence of the AMV-Eggplant-EG isolate was registered under GenBank accession number MW428250.

### 4.4. Multiple Alignments and Phylogenetic Analysis

The AMV-Eggplant-EG sequence generated in this study was compared with available AMV sequences in GenBank (http://www.ncbi.nlm.nih.gov/BLAST/, accessed on 1 December 2020). Multiple alignments of sequences were performed using BioEdit software (Ver.7.2.5), and ClustalW was included within the software MEGA 7.0. The phylogenetic reconstructions were performed using the maximum parsimony method implemented in MEGA 7.0 with a statistical confidence of 1000 replicates to assess the constructed phylogenetic tree’s reliability.

### 4.5. Plant Materials, Eggplant Treatments, and Alfalfa mosaic virus Inoculation

Eggplant (*Solanum melongena*) seeds were grown in plastic pots in a mixture of sand and clay (1:2 *v*/*v*) in separate growth chambers with a photoperiod of 12 h. The temperatures in the light and dark period were 27 °C and 23 °C, respectively; the relative humidity was about 70%. The plants had a relative water content of 100%. After seven days of growth, seedlings were transferred to 40 cm pots containing a sterile soil mixture of 35% clay, 35% sand, and 30% peat moss and grown under the same conditions. The plants were divided into six groups after 14 days of growth. Five replications were made of each group. The groups were divided into:

1. The first group, the plants were inoculated with only phosphate buffer without virus as mock inoculation (MK).
2. The second group, the plants were foliar sprayed with 100 µM melatonin (MLT), combined with two drops of Tween 80 into the leaves until run-off.
3. The third group, the plants were foliar sprayed with 100 µM salicylic acid (SA), combined with two drops of Tween 80 into the leaves until run-off.
4. The fourth group, the plants were inoculated with *Alfalfa mosaic virus* as challenge control (ChC).
5. The fifth group, the plants were foliar sprayed with 100 µM MLT and then inoculated after three days with AMV.
6. The sixth group, the plants were foliar sprayed with 100 µM SA and then inoculated after three days with AMV.

All the leaves were inoculated mechanically. The inoculation was prepared from infected leaves ground in a mortar containing a phosphate buffer of 0.1 M (pH 7.0). It was filtered through two layers of cheesecloth. The leaves of healthy plants were dusted with carborundum and gently rubbed with a soft cotton swab that had previously been dipped into an inoculum suspension. The percentage of infected plants and the severity of symptoms were assessed three weeks after inoculation using the following rating scale: 0 = no symptoms, 2 = mild chlorosis, 4 = mottling, net yellow, blisters and leaf narrow, 6 = leaf distortion, vein enation, and necrotic intervention. Disease severity values were calculated using the formula of Yang, et al. [90].

$$\mathrm{DS}\;\left(\%\right)=\frac{\sum \left(\mathrm{Disease}\;\mathrm{grade}\;\times \;\mathrm{Number}\;\mathrm{of}\;\mathrm{plants}\;\mathrm{in}\;\mathrm{each}\;\mathrm{grade}\right)}{\left(\mathrm{Total}\;\mathrm{number}\;\mathrm{of}\;\mathrm{plants}\;\times \;\mathrm{Highest}\;\mathrm{disease}\;\mathrm{grade}\right)}\times 100$$

### 4.6. Growth Indices

Ten plants were harvested randomly from each treatment to determine the shoot and root length, number of leaves, leaf surface, and leaf biomass three weeks after inoculation.

### 4.7. Biochemical Analysis

Three weeks after inoculation, the younger leaves produced from both the control and treatment plants were collected for examining biochemical changes.

#### 4.7.1. Determination of Photosynthetic Pigments

Fresh leaves (0.5 g samples) were ground with acetone (80%), and the homogenate was filtered through Whatman No 1 filter paper. A spectrophotometer was used to read the absorbance of filtrate at 470, 652, and 665 nm to assess the chlorophyll-a, chlorophyll-b [91], and carotenoids [92].

#### 4.7.2. Determination of Oxidative Damage Marker

A half gram of eggplant leaf was ground with a KH_2_PO_4_-KOH buffer (pH 7.8) and centrifuged at 10,000× *g* for 15 min. The collected supernatant was used to determine H_2_O_2_ using TiCl_2_ and a spectrophotometer was used to read the mixture’s absorbance at 410 nm according to an earlier protocol by Patterso, et al. [93]. The supernatant was also used to determine O_2_^−^ by adding it to hydroxylamine hydrochloride and heating at 25 °C for 1 h, it was then mixed with sulfanilamide and α-naphthylamine at 25 °C for 20 min. The mixture was read at 530 nm using a spectrophotometer [94]. In addition, the supernatant was used to determine OH by adding 100 μM of FeCl_3_, 104 μM of EDTA, one mM of H_2_O_2_, and 100 μM of ascorbate to the final 1 mL and heating at 37 °C for 1 h. The absorbance was read at 532 nm using a spectrophotometer, according to Babbs et al. [95].

The MDA level was determined by using 0.5 g of leaf samples ground in 5 mL of 0.1% (*w*/*v*) trichloroacetic acid, and centrifuged at 10,000× *g* for 10 min, and then 5 mL of 20% TCA containing 0.5% (*w*/*v*) 2-thiobarbituric acid was added to 2 mL of the supernatant. The mixture was heated at 95 °C for 30 min, then cooled, and the mixture’s absorbance was read at 532 and 600 nm using a spectrophotometer as described by Heath and Packer [96].

#### 4.7.3. Determination of Total Phenolic and Flavonoid Compounds

First, 80% methanol (*v*/*v*) was homogenized with 0.5 g of dried leaves and centrifuged for 20 min at 10,000× *g* to obtain a clean solution. For estimating the total phenolic compounds, the Folin–Ciocalteu (FC) test was used, and the spectrophotometer read the absorption at 765 nm as described by Singleton and Rossi [97]. The methanolic extract was also used to determine flavonoid compounds by using an ALCl_3_ reagent. The obtained color has been measured at 510 nm using a spectrophotometry method described by Zhishen et al. [98].

#### 4.7.4. Determination of Lignin

One gram of dry leaves was ground in ice-cold 95% ethanol, and the homogenate was centrifuged at 3000× *g* for 15 min. The resulting pellet was washed with 95% ethanol three times and with a 1:2 (*v*/*v*) hexane/ethanol mixture twice and dried overnight at 37 °C. In acetic acid, the dried pellet was ultrasonically homogenized and centrifuged at 1000× *g* for a total of 5 min. After heating at 70 °C for 30 min, the sample was applied with 270 mL of 2 M NaOH, 30 mL of 7.5 M of hydroxylamine, and 900 mL of acetic acid. Samples were centrifuged for 5 min at 1000× *g*, and the lignin content was read at 280 nm, according to Bruce and West [99].

#### 4.7.5. Determination of Salicylic Acid

One gram of the leaf tissue was homogenized in 2.5 mL of 90% methanol and centrifuged for 15 min at 12,000× *g*. The pellet was homogenized again with 100% methanol (*v*/*v*) and centrifuged for an additional 15 min at 12,000× *g*. The supernatants were mixed and dried under liquid N_2_ from both extractions. In 2.5 mL of 5% trichloroacetic acid (*v*/*v*), the residue was resuspended and filtered. Filtration was separated by 5 mL (1:1 *v*/*v*) of a 1% (*v/v*) isopropanol mixture of ethyl acetate/cyclohexane. SA was determined using HPLC, as described by Raskin et al. [100].

#### 4.7.6. Enzymatic Antioxidant Assays

Half a gram of fresh leaf was ground with 10 mL of 50 mM KH_2_PO_4_ buffer (pH 7.8), and the sample was centrifuged at 10,000× *g* for 15 min at 5 °C. Then the protein concentration of the extract was determined [101]. Superoxide dismutase activity (SOD, EC 1.15.1.1) was measured by the Kono [102] method with the help of Na_2_CO_3_ as a buffer and nitrobluetetrazolium (NPT) as the substrate. The inhibition was reported using a spectrophotometer at 540 nm of NBT reduction. A catalase activity test (CAT, EC 1.11.1.6) was carried out using potassium phosphate as a buffer, with H_2_O_2_ as a substrate as described by Aebi [103], and the absorption was read at 240 nm. The peroxidase (POX, EC 1.11.1.7) activity test was performed by the method reported in Thomas et al. [104] using benzidine and a spectrophotometer to record the absorbance at 470 nm. The phenylalanine ammonia-lyase activity (PAL, EC 4.3.1.5) was assessed as described by Assis et al. [105].

#### 4.7.7. RT-PCR Analysis

Four days after inoculation, the leaf tissues were sampled from inoculated eggplants treated with MLT and SA, and challenge control plants. The protocol was defined by Wang et al. [106] and, Derbalah and Elsharkawy [107]. qRT-PCR was performed with gene-specific primers [108], as shown in Table 3. Standardizing, the ACTIN constitutive gene’s abundance was achieved to normalize the target gene quantity (Table 3). The 7000 RT-PCR system was used, and the data collected were analyzed with the ABI PRISM 7000 program (Bio-Rad iCycler).

### 4.8. Statistical Analysis

The experimental design was randomized, and statistical analysis was conducted with the statistical software SPSS (Statistical Package for the Social Science Version 26.0) [109]. A one-way or, two-way ANOVA with post hoc test variance analysis from Fisher’s test with Levene’s sample parametric distribution, was used for the quantitative analysis. The confidence interval was set to 95%, and the negotiated margin for error was fixed at 5%.

## 5. Conclusions

AMV Egyptian isolate (AMV-Eggplant-EG) was biologically isolated from the six positive samples tested by DAS-ELISA and from the similar local lesions induced on *Chenopodium amaranticolor,* and then it was re-inoculated in healthy *Solanum melongena* as a source of AMV-Eggplant-EG and confirmed by DAS-ELISA. RT-PCR assay with a pair of primers specific for coat protein (CP) encoding RNA 3 of AMV yielded an amplicon of 666 bp from infected plants of *Solanum melongena* with AMV-Eggplant-EG. The amplified PCR product was cloned and sequenced. Analysis of the AMV-Eggplant-EG sequence revealed 666 nucleotides (nt) of the complete CP gene (translating 221 amino acid (aa) residues). Analysis of phylogeny was performed for nt and deduced aa sequences of the CP gene using the maximum parsimony method clustered AMV-Eggplant-EG in the lineage of Egyptian isolates.

**Figure 13:**
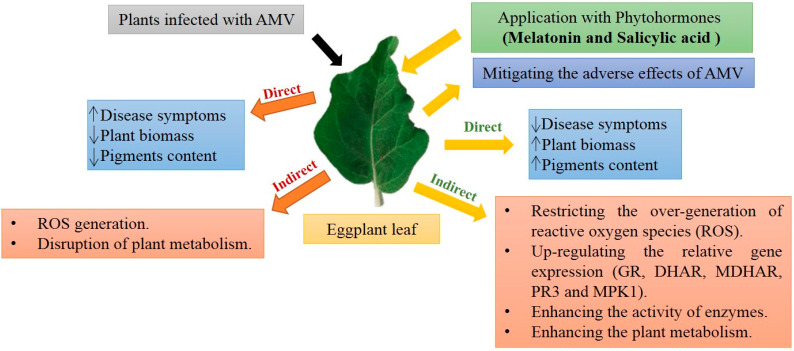
Possible mechanism of MLT and SA to resist AMV infection in eggplants.

Eggplants can resist AMV infection through a wide range of cellular processes: (i) up-regulation of different genes; (ii) changes in pathway levels of various compounds, like reactive oxygen species (ROS); (iii) stimulation of various transcription factors activity, and regulation of defense genes; (iv) stimulation of protective signaling enzymes and phytohormones such as SA. The results indicated that MTL and SA induced systemic acquired resistance and could regulate ROS production, thus contributing to enhanced resistance of eggplants to AMV. Since MLT is considered a naturally occurring and safe compound, MLT and SA’s application could be an ecofriendly strategy for the effective management of AMV infection in eggplants by reducing the virus concentration. In conclusion, foliar spraying of challenge control eggplants with MLT and SA could be a method for counteracting AMV infection by activating phenolic and flavonoids’ accumulation, lignin content, endogenous SA content, the antioxidant defense system, genes expression and molecules involved in scavenging free radicals (Figure 13).

## Figures and Tables

**Table 1:** Comparison of coat protein gene sequence identity: AMV-Eggplant Egyptian isolate sequence is compared at nucleotide (nt) and amino acid (aa) levels using BLAST and DiAlign tools with *Alfalfa mosaic virus* isolates available in GenBank.

GenBank Accession No.	Origin	Year	Host	Strains	Identity	
					nt (666)	aa (221)
LN846978	Egypt	2015	Tomato	shark-EG	644/666 (96.70%)	211/221 (95.48%)
HQ288892		2010	*S. tuberosum*	Potato-EG	644/666 (96.70%)	210/221 (95.02%)
LN846979		2015	Tomato	mans-EG	643/666 (96.55%)	210/221 (95.02%)
KY471416		2014	*S. tuberosum*	CP2-EG	638/668 (95.51%)	204/221 (92.31%)
KY549685		2014	-	FRE-EG	638/668 (95.51%)	204/221 (92.31%)
MH625710		2016	Basil	Basil-EG	619/666 (92.94%)	198/221 (89.59%)
HQ185569	USA	2006	Soybean	Joe Davis	643/666 (96.55%)	210/221 (95.02%)
M59241		1991	Lucerne	YSMV	638/666 (95.80%)	209/221 (94.57%)
K02703		1983	*N.tabacum*	425M	639/666 (95.95%)	208/221 (94.12%)
JN256026		2011	Soybean	SE12	639/666 (95.95%)	208/221 (94.12%)
L00162		1977	*N. glutinosa*	425L	635/666 (95.35%)	205/221 (92.76%)
AJ130709	France	1998	Wild tomato	Lyh1	634/666 (95.20%)	204/221 (92.31%)
AJ130708		1998	Carrot	Dac16	634/666 (95.20%)	204/221 (92.31%)
AJ130707		1998	Pepper	Caa1	636/666 (95.50%)	203/221 (91.86%)
AJ130703		1998	Tomato	Lye80	629/666 (94.44%)	201/221 (90.95%)
Y09110	Italy	1997	Tomato	Danza	641/666 (96.25%)	209/221 (94.57%)
AJ130706		1998	Bean	F430	639/666 (95.95%)	209/221 (94.57%)
AF015716	England	1997	Garden lupin	VRU	634/666 (95.20%)	205/221 (92.76%)
AF015717		1997	Garden lupin	15/64	628/666 (94.29%)	204/221 (92.31%)
DQ314753	Canada	2004	Potato	Ca401	637/661 (96.37%)	209/220 (95.00%)
DQ314751		2004	Potato	Ca399	642/667 (96.25%)	209/221 (94.57%)
DQ314749		2004	Potato	Ca375	640/667 (95.95%)	209/221 (94.57%)
DQ314752		2004	Potato	Ca400	640/667 (95.80%)	209/221 (94.57%)
DQ314750		2004	Potato	Ca175	639/667 (95.80%)	208/221 (94.12%)
DQ314754		2004	Potato	Ca508	636/667 (95.35%)	207/221 (93.67%)
JX112759	Australia	2001	*Medicago sativa*	Hu	641/666 (96.25%)	209/221 (94.57%)
HM807304		1985	*Medicago sativa*	N20	638/666 (95.80%)	209/221 (94.57%)
AF294433	Korea	2000	*Solanum tuberosum*	KR2	641/666 (96.25%)	211/221 (95.48%)
AF294432		2000	*Solanum tuberosum*	KR1	638/666 (95.80%)	208/221 (94.12%)
AF215664	New Zealand	1999	*S. tuberosum*	NZ34	640/666 (96.10%)	208/221 (94.12%)

**Table 2:** Effect of foliar spraying of MLT and SA on virus concentration, percentage of infection (%), and disease severity of eggplant leaves under AMV infection.

Treatments	Virus Concentration	Percentage of Infection (%)	Disease Severity (%)
Challenge control (ChC)	0.50 a	100 a	90.0 a
Melatonin + V	0.18 c	25 c	13.33 c
Salicylic acid + V	0.22 b	40 b	21.66 b

According to Fisher’s test, the different letters (a, b, c) are significantly different at the treatments’ 0.05 level.

**Table 3:** Forward and reverse primers sequence for GR, DHAR, MDHAR, PR3, MPK1, and ACTIN genes.

Gene	Forward Primer (5′–3′)	Reverse Primer (5′–3′)
GR Glutathione reductase	TTGGTGGAACGTGTGTTCTT	TCTCATTCACTTCCCATCCA
DHAR Dehydroascorbate reductase	GAAGTGGAGTGTGCCTGAAA	CGTACTTCTCTTCAGCCTTGG
MDHAR Monodehydroascorbate reductase	TCCGAACAAACATACCTGGA	GTGTGCGTGTGTGCAGTTAG
PR3 Chitinase expression	AGAGAACAAGGTAGCCCAGG	TAAAAGGTCCACTCCGATGGC
MPK1 Mitogen-activated protein kinase 1	CCTCCGTGGGTTGAAATAC	GTCACAACATATTCGGTCATAAAG
ACTIN	TGGTCGGAATGGGACAGAAG	CTCAGTCAGGAGAACAGGGT
